# Analysis of the Effectiveness of Technological Lubricants with the Addition of Boric Acid in Sheet Metal Forming

**DOI:** 10.3390/ma16145125

**Published:** 2023-07-20

**Authors:** Janina Adamus, Wojciech Więckowski, Piotr Lacki

**Affiliations:** 1 Department of Civil Engineering, Faculty of Civil Engineering, Czestochowa University of Technology, 69 Dąbrowskiego St., 42-201 Częstochowa, Poland; piotr@lacki.com.pl; 2 Department of Technology and Automation, Faculty of Mechanical Engineering and Computer Science, Czestochowa University of Technology, 69 Dąbrowskiego St., 42-201 Częstochowa, Poland; wojciech.wieckowski@pcz.pl

**Keywords:** sheet metal forming, ecological lubricant, boric acid, aluminum, steel

## Abstract

One of the main problems during sheet metal forming is the reduction in coefficient of friction and separation of contact surfaces in order to eliminate buildups of the formed material on the forming tools. For this purpose, technological lubricants based on mineral or synthetic oils are usually used. Unfortunately, their removal from the drawn parts and their subsequent utilization pose many problems and are expensive. Environmentally benign lubricants based on vegetable oils with the addition of boric acid could be an effective alternative to lubricants based on mineral and synthetic oils; however, the solubility of boric acid in oils is limited. Therefore, the paper proposes new, effective, and environmentally friendly methods for applying boric acid to the metal sheet by spraying it on a thin rapeseed oil layer previously applied to the metal sheet or by spraying a 25% solution of boric acid in methyl alcohol onto the sheet. The effectiveness of such lubrication was assessed on the basis of the so-called strip drawing test, Erichsen cupping test, and formation of cylindrical drawn parts in industrial conditions. The tests showed that the addition of boric acid was most effective for forming the DC01 steel sheet, reducing the coefficient of friction by about 60% compared to base oil lubrication. Although its usefulness is lower in the case of other frictional pairs, it eliminates the phenomenon of the formed material sticking to the tool, thus extending the life of the forming tools. The use of the proposed solution reduces production costs and indirectly boosts environmental protection. Moreover, an explanation of the tribological mechanism contributing to the lubrication action of boric acid is given.

## 1. Introduction

The progressive degradation of the natural environment is closely related to human industrial activity, which results in large amounts of undesirable substances getting into the atmosphere, soil, and water. It is especially visible in large urban agglomerations. The environmental pollution negatively impacts health, food quality, and crop yields. Minimization of environment degradation is one of the main ecological challenges of the 21st century. To meet this challenge, it is necessary to constantly raise environmental awareness. Cooper et al. [1,2], analyzing sheet metal forming processes, found that they are quite expensive and energy-intensive, and that there is still much room for improving their potential energy and material efficiency. Sheet metal forming processes, like all other mass or large-scale production processes, should be evaluated in terms of their environmental impact. From the tribological perspective, the goal is to reduce large amounts of grease wastes containing environmentally hazardous active elements such as chlorine, sulfur, or phosphorus. Research should focus on the possibility of sheet metal forming without lubrication or with the use of environmentally friendly lubricants. Therefore, the authors of this work undertook an initiative aimed at reducing the amount of environmentally hazardous lubricant waste generated in sheet metal forming.

Sheet metal forming is one of the most popular techniques for producing light and durable products [1,2,3,4]. In this way, components ranging from small metal accessories and everyday items to large components for the automotive, aviation, and space industries are produced. Typically, the surface of the drawn part is 2–5 times larger than that of the blank, revealing new, non-oxide-coated sheet metal surfaces. This leads to a direct contact of the formed metal surface (“in situ”) with the tool working surface, with relatively high pressures, ranging from 1 to 100 MPa. The size of the pressures depends mainly on the type of formed material, the sheet thickness, and the type of operation. In order to prevent direct contact of the deformed material with the tool, technological lubricants are used. They are applied directly before the forming operation to the tool or sheet. Until recently, steel products made of drawing or deep-drawing sheets dominated; however, with the growing tendency to reduce the weight of the structure and increase its corrosion resistance, other materials, such as stainless steel, aluminum, and titanium, as well as their alloys, were introduced. Forming these sheets requires intensive lubrication to facilitate the material flow and eliminate the phenomenon of sticking of the formed material to the tool’s working surfaces [5,6,7,8,9]. Lubricants based on mineral and synthetic oils, as well as water-based lubricants are mostly used. Less often, solid PVC (polyvinyl chloride) and polyurethane or molybdenum disulfide and graphite [10] films are used. Technological lubricants for sheet metal forming based on mineral and synthetic oils have good lubricating properties and a wide range of viscosity. Thanks to the possibility of modifying their properties with various additives, they can be adapted to various operating conditions. Mineral oil-based lubricants are relatively cheap and readily available compared to synthetic oil-based lubricants. However, they have a more adverse impact on the natural environment. They often contain harmful substances such as chlorine derivatives and sulfur compounds. These lubricants are difficult to biodegrade. Water-based technological lubricants dissipate heat from the frictional pair much better and are easier to remove from the drawn parts, but their lubricating properties are worse than mineral and synthetic oils, which limits the scope of their use and protection against corrosion. They have a greater tendency to flow out of the frictional pair, which requires more frequent application and the introduction of thickening agents. Recently, scientists have evaluated technological lubricants in terms of their positive effect on sheet drawability, while also taking into account their impact on the environment [11,12,13]. According to [9], the problem is that, unlike machine lubrication, where the lubricant works in a closed circuit, metal forming involves a so-called open friction node, which results in a much higher consumption of technological lubricant. In sheet metal forming processes, a new portion of lubricant must be introduced into a friction node after each operation. A large part of it is irretrievably lost, i.e., it is “picked up” by the deformed material and remains on the drawn-part surfaces until the next operation of surface treatment, such as the application coatings, including varnishes. To minimize hazardous lubricant wastes, some researchers [14,15,16,17,18,19] suggested replacing existing technological lubricants with lubricants based on vegetable oils. Bartz [20] stated that, for petroleum-based oils, the limiting value of biodegradability is around 30–65%, whereas, for biobased oils, it is around 95%. Apart from the fact that vegetable oils are biodegradable substances, due to the presence of long fatty acid chains and the ability to adhere to metal surfaces, they provide good lubrication under boundary friction conditions [21,22]. However, as noted by Deshmukh et al. [23], vegetable oils degrade at elevated temperatures, which limits their use only to cold forming processes. Among the disadvantages of vegetable oils, Chowdary [24] also mentioned bacterial deterioration, solidification at low temperatures, and inferior thermal oxidation stability.

To improve the lubricating properties and ensure stable lubricant function, different additives are applied to the base oils [25]. Graphite is one of the earliest used liquid lubricant additives. Its application initiated research into materials with a similar crystal structure, now known as two-dimensional (2D) materials [26,27]. Examples of 2D materials are MoS_2_ [10,28,29], h-BN [18,30,31], and boric acid [15,32], which as Rao and Xie [32] underlined, have a strong tendency to form chemical bonding films on the oxidized surface of aluminum. The specific layered structure of these materials ensures low frictional resistance. Strong covalent bonds of atoms making up individual layers and weak interlayer interactions due to van der Waals forces ensure easy sliding of layers in relation to each other, reducing friction. Most of these materials have superlubricity properties, which means that the coefficient of friction (*CoF*) is less than 0.01 [33,34,35,36,37]. Studies on superlubricity are promising due to the possibility of achieving energy savings and increasing the durability of various mechanisms and tools, including forming dies. Research has also been conducted to improve the tribological properties of oils by introducing nanoparticles of metal oxides. Kumar and Gautam [16] tested the tribological performance of vegetable (sunflower and soybean) oils with CuO and ZrO_2_ nanoparticle additives. When separately added, CuO nanoparticles improved antifriction behavior, whereas ZrO_2_ nanoparticles improved anti-wear behavior. The simultaneous action of CuO and ZrO_2_ nanoparticles improved both the antifriction and anti-wear behavior of the oils.

Special extreme pressure (EP) and anti-wear (AW) additives are used when sheet metal forming requires high pressure. Although traditional EP and AW additives (phosphorus, sulfur, or chlorine compounds) are considered to be extremely harmful to the environment, according to Li et al. [38], some EP and AW nanoparticle additives have operational properties comparable to the traditional additives, while being environmentally friendly. The action of traditional EP and AW additives is mainly based on adsorption or a chemical reaction with the friction surface of the metal, while the mechanism of the new EP and AW green nanoparticles mainly includes a repairing effect, polishing effect, protective film formation mechanism, and rolling bearing effect.

To reduce the amount of lubricant waste, some studies explored so-called minimum quantify lubrication (MQL) technologies [39].

The authors of this study have continued their research on lubricants based on rapeseed oil with addition of boric acid. Given the poor solubility of boric acid in water and oils [40], the authors propose two methods of applying boric acid to the sheet: firstly, by spraying finely powdered boric acid on a layer of lubricant previously applied to the sheet according to the patent [41]; secondly, a new method of spraying the sheet with a 25% solution of boric acid in methyl alcohol. After the alcohol evaporates, a thin smooth layer of boric acid remains on the surface of the sheet, which is considered harmless to the environment. Due to its antiviral, antifungal, and antiseptic properties, boric acid is used in medicine. Moreover, it can be easily removed from the sheet surface with a stream of running water. The second method, in particular, allows for avoiding the problems related to the removal of lubricant from the drawn part surface, a problem which is particularly noticed by companies applying coatings on drawn parts. It is well known fact that the presence of even a small amount of technological lubricant weakens the adhesion of coatings to the substrate, and their effective and environmentally friendly removal is still a great challenge.

## 2. Goal and Range of Tests

The main goal of this research is to find an effective method of applying boric acid to metal sheets prior to forming. The choice of boric acid as a lubricating substance resulted from the fact that its crystal structure is similar to that of graphite, which has been used for years as a solid lubricant or additive to lubricants. Both graphite and boric acid show a layered crystal structure characterized by weak bonds between the layers and strong interactions between the atoms located in the horizontal layers.

Although graphite significantly reduces the coefficient of friction, it builds a layer of dirt on the surface of products that is difficult to remove. Therefore, attempts have been made to replace graphite with other substances that can be easily removed from the surface of drawn parts. This study analyzes lubricants with the addition of boric acid, which can be in the form of colorless crystals or a white crystalline powder.

**Figure 1:**
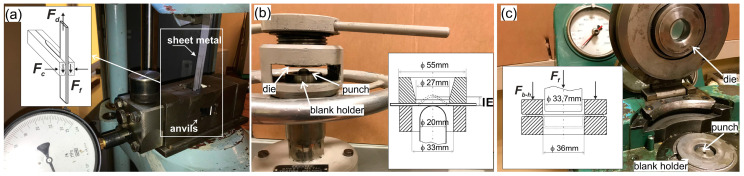
Experimental tests: (**a**) strip drawing test (*F_d_*—drawing force, *F_c_*—clamping force, *F_f_*—frictional force), (**b**) Erichsen cupping test, (**c**) deep drawing of cylindrical cup (*F_f_*—forming force, *F_b-h_*—blank holder force).

The lubricating effectiveness was assessed on the basis of the following methods:

- The so-called strip drawing test [42], which consists of drawing a sheet metal strip between two flat counter samples in presence of the tested lubricant (Figure 1a),
- The Erichsen cupping test according to the standard PN-EN ISO 20482:2004 [43] (Figure 1b),
- The deep drawing of the cylindrical cup with diameter of 35 mm (Figure 1c).

The following sheets were analyzed: EN AW-1050A (according to [44]) aluminum alloy sheet with a thickness of 1.0 mm, EN AW-2017A (according to [44]) aluminum alloy sheet with a thickness of 1.0 mm, EN DC01 (according to [45]) cold-rolled low-carbon steel sheet with a thickness of 0.9 mm, and EN X5CrNi18-10 (according to standard [46]) austenitic chromium–nickel stainless-steel sheet with a thickness of 1.0 mm.

The mechanical properties of the analyzed sheets are given in Table 1.

The strip drawing test was performed on a tensile testing machine EU-20 using the special device shown in Figure 1a. During the test, 20 mm wide and 500 mm long sheet strips were drawn between two counter samples (anvils) with a speed of 50 mm/min. The anvils were made of NC10 tool steel (with chemical composition: C—1.5–1.8, Si—0.15–0.4, Mn—0.15–0.45, P—max. 0.03, S—max 0.03, and Cr—11–13 according to standard [47]). The hardness of the counter-samples was 60 HRC. The working surfaces of some counter-samples were prepared by grinding (Ra ≈ 0.28 µm, Rz ≈ 2.2 µm), and those of others were prepared by polishing (Ra ≈ 0.02 µm, Rz ≈ 0.1 µm). The strips were cut out from the analyzed sheets in a direction perpendicular to the rolling direction. The longer edges of the cut sheet strips were deburred. The surface roughness of the samples (strips) is summarized in Table 2.

The coefficients of friction (*CoF*s) were determined for the technically dry friction and in the presence of the following lubricants:

- Rapeseed oil—lubricant No. 1,
- Rapeseed oil with boric acid additive applied to the sheet surface according to the patent [41]—lubricant No. 2,
- 25% boric acid solution in methyl alcohol—lubricant No. 3.

Before applying lubricants to the sheet metal strips, their surfaces were degreased with extraction gasoline. Lubricants were applied to the sheet strips by spraying.

**Table 2:** Surface roughness of the samples.

Material	Roughness Parameter	
	Ra, µm	Rz, µm
EN AW-2017A	~0.22	~1.4
EN AW-1050A	~0.35	~2.5
EN DC01	~1.43	~8.5
EN X5CrNi18-10	~0.15	~1.2

During the strip drawing test, the clamping force *F_c_* and the drawing force *F_d_* were registered (see Figure 1). The coefficient of friction was calculated as follows:

(1) $$CoF=\frac{F_{d}}{2Fc},$$

where *F_d_* is the drawing force, and *F_c_* is the clamping force.

Three tests were performed for each frictional pair and lubrication conditions, from which the average CoF values were calculated. 

While the Erichsen cupping test is typically used to evaluate sheet drawability and adhesion of coatings to sheets, the test was used herein to evaluate the effectiveness of lubricants. In the Erichsen cupping test, a spherical punch with diameter of 20 mm was pressed into the square sheet blank with dimensions of 65 × 65 mm. The blank was clamped between the blank holder and the die. The speed of pressing the punch into the sheet was 10 mm/min. As the blank could not slide out from the blank holder, the spherical cups were formed only by stretching. In this test, the lubricant effectiveness was assessed on the basis of the cup depth at the moment of its fracture, which was denoted as the Erichsen index (IE) in mm. As in the strip drawing test, before the tests, the sheet blank surfaces were degreased with extraction gasoline, and then the lubricant was applied to the sheet blanks from the punch side.

Three tests were performed for each analyzed sheet metal and lubrication conditions, the test result is the average value of IE.

Forming of cylindrical cups was carried out in a pressing plant, i.e., in industrial conditions, on a double-action deep drawing hydraulic press, using a production set of tools. During forming, the blank holder force was selected so that the sheet could slide out from under the blank holder, without causing sheet wrinkling. Thus, the bottom of the drawn part was formed by stretching, and the cylindrical wall was formed mainly by drawing. The cylindrical cups were formed from a sheet metal disc with a diameter of 55 mm. As in the laboratory tests, i.e., in the strip drawing test and in the Erichsen cupping test, before forming, the surfaces of the sheet blanks were degreased with extraction gasoline. Since the tools were used in mass production, in order to eliminate the influence of previously used technological lubricants, before starting the forming process, the die, punch, and blank holder were thoroughly washed. This time, the lubricants were applied to the blank holder and die. The lubricant effectiveness was assessed on the basis of the value of forming force measured during forming process.

Three tests were performed for each analyzed sheet metal and lubrication conditions, the average values of the forming force were given as the result.

## 3. Results and Discussion

**Figure 2:**
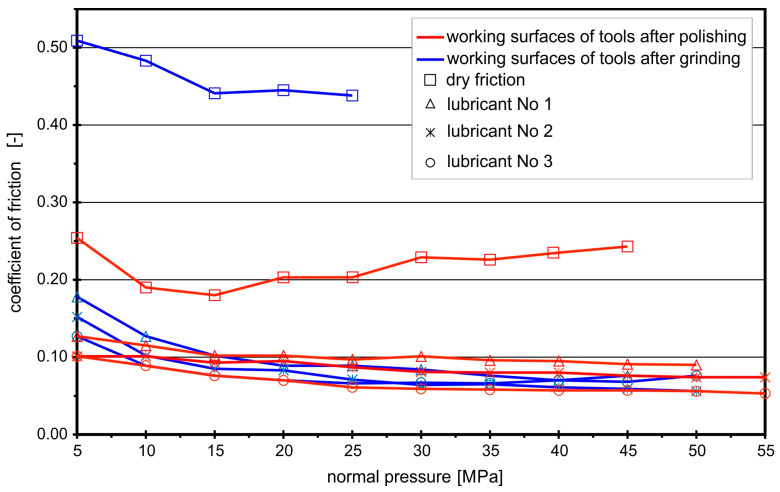
Coefficients of friction for contact pair “EN AW-2017A–steel NC10”.

**Figure 3:**
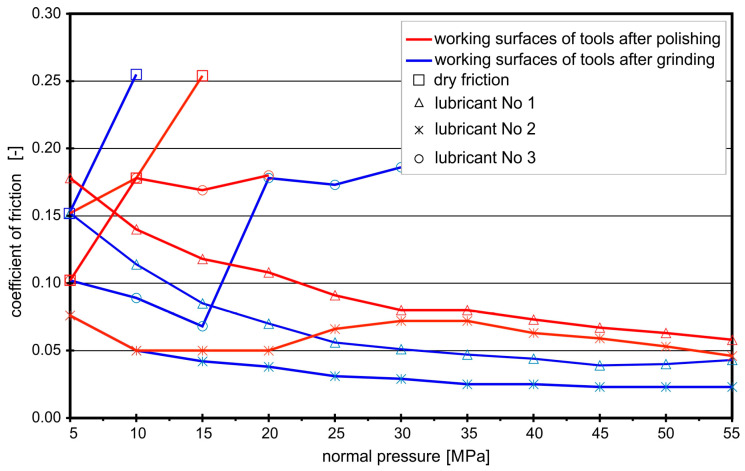
Coefficients of friction for contact pair “EN AW-1050A–steel NC10”.

**Figure 4:**
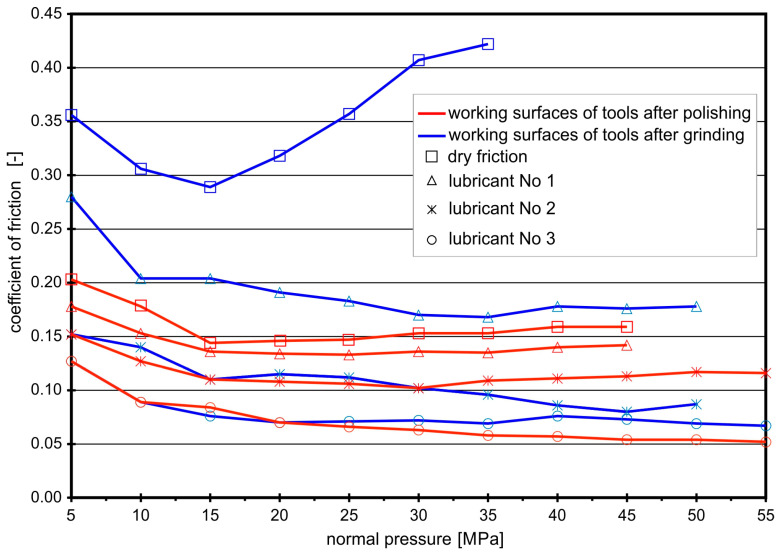
Coefficients of friction for contact pair “steel EN DC01–steel NC10”.

**Figure 5:**
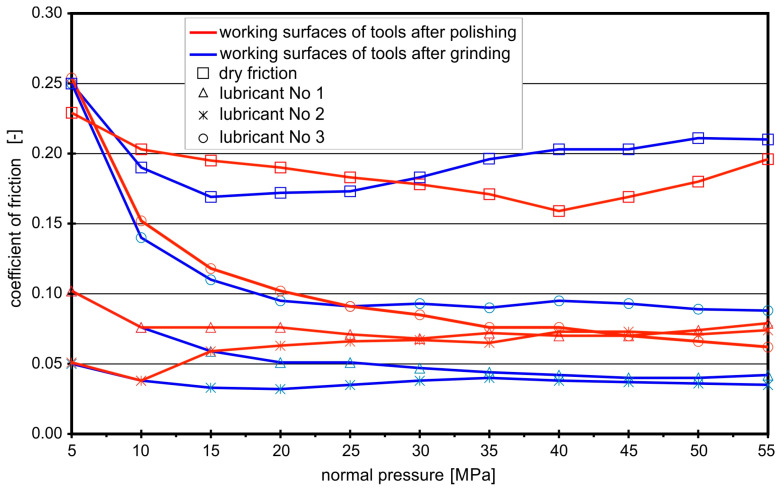
Coefficients of friction for contact pair “steel EN X5CrNi18-10–steel NC10”.

The coefficients of friction for the analyzed contact pairs “aluminum–steel” and “steel–steel”, under the conditions of technically dry friction and in the presence of the tested lubricants, determined in the strip drawing test, are shown in Figure 2, Figure 3, Figure 4 and Figure 5. The coefficients of friction for the frictional pair “EN AW-2017A–steel NC10” are presented in Figure 2.

In the case of the contact pair “EN AW-2017A–steel NC10”, tested lubricants effectively reduced the coefficients of friction for both ground and polished working surfaces of the tool. Under conditions of technically dry friction, the average *CoF* value was about 0.45 for the ground working surfaces of the tool and within the range 0.20–0.25 for the polished ones. The use of lubricants, even rapeseed oil without any additives (lubricant No. 1), significantly decreased frictional resistance. The average *CoF* for the polished surfaces in the presence of rapeseed oil was 0.07, and that for the ground surfaces was 0.09. After exceeding the normal pressures of 50 MPa, the oil film was broken. Although the use of boric acid as an additive to rapeseed oil or dissolved in methyl alcohol did not result in further significant reduction in the coefficients of friction (*CoF* = 0.05–0.07), the durability of the lubricating films increased and, despite the increase in normal pressure, they protected better working surfaces against seizure.

The friction process was slightly different when drawing the EN AW-1050A aluminum sheet, which is much more plastic than the EN AW-2017A sheet (Figure 3).

Drawing the strips of EN AW-1050A aluminum sheet under the condition of technically dry friction caused almost immediate seizure of the contact surfaces, which was manifested by a rapid increase in the drawing force and an increase in the adhesion of aluminum to steel anvil surfaces, regardless of the surface treatment method, i.e., regardless of whether they were ground or polished. The lowest values of *CoF* in the entire range of the tested normal pressures were obtained for No. 2 lubricant, i.e., boric acid sprayed on the layer of rapeseed oil applied to the sheet having contact with ground anvils. No. 3 lubricant provided a low coefficient of friction only at low normal pressures. In the range of normal pressures below 15 MPa, lower values of the coefficient of friction in the case of ground surfaces (*CoF* ≤ 0.1) compared to polished surfaces (*CoF* ≥ 0.15) were due to the fact that the working surfaces of the ground anvils were rougher and allowed the formation of lubricating micropockets filled with boric acid particles. Therefore, the friction took place in boric acid such that a lower *CoF* was obtained. However, with an increase in normal pressures above 15 MPa, in the case of ground anvils, the friction coefficient increased sharply, and, in the case of polished anvils, the lubricating film was broken. When drawing the EN AW-1050A aluminum sheet strip, it was observed that the sprayed layer of boric acid dissolved in methyl alcohol peeled off and detached from the sheet surface, as the sheet became plastic and deformed (stretches) with the increase in drawing force. The exposed surfaces of the sheet adhered to the anvil surfaces, causing an increase in the frictional resistances.

A more pronounced influence of boric acid on the value of the coefficients of friction was observed for the “steel EN DC01–steel NC10” contact pair (Figure 4).

When drawing EN DC01 steel strips between ground anvils with no lubrication, the first signs of seizure appeared when the normal pressure exceeded the value of 15 MPa. With the increase in pressure, the frictional resistance increased intensively. The introduction of rapeseed oil between the contacting surfaces lowered the coefficient of friction to a value of about 0.18. A further reduction in the *CoF* was obtained after the introduction of boric acid.

When drawing EN DC01 steel strips between the polished anvils, in conditions of technically dry friction, the coefficient of friction remained at the level of approximately 0.15. Rapeseed oil only slightly decreased the coefficient of friction (*CoF* = 0.14). The addition of boric acid to rapeseed oil resulted in a reduction in *CoF* to a value in the range of 0.08–0.12. The lowest *CoF* values were obtained when lubricating with 25% boric acid solution in methyl alcohol, with slightly higher values obtained for the ground working surfaces (*CoF* = 0.07).

The analysis of the friction coefficients for the contact pair “steel EN X5CrNi18-10–steel NC10” (Figure 5) shows how much surface roughness affected their values. The lowest *CoF* values were obtained when the sheet was drawn between anvils with ground surfaces and lubricated with rapeseed oil with the addition of boric acid. At that time, there were conditions for the formation of lubricating micropockets, which made it easier for the sheet metal strip to slide on the tool surfaces. Lubricant No. 3 was the least effective, especially for the polished anvils. There were no conditions for the emergence of lubricating pockets on the smooth surface. In the case of ground surfaces, the *CoF* decreased with the increase of normal pressures, which can also be explained by the action of lubricating micropockets. Relatively high *CoF* values at low normal pressures resulted from the low roughness of the EN X5CrNi18-10 sheet (Ra ≈ 0.15 µm, Rz ≈ 1.2 µm; the lowest among the analyzed sheets), which limited the adhesion of the lubricating coating, formed after alcohol evaporation, to the sheet. Only increasing the normal pressures was conducive to filling the micropockets with lubricant on the surfaces of ground anvils and transferring the friction to the layer of boric acid. The average *CoF* for the ground anvils in the presence of lubricant No. 3 was of 0.07, and that for the polished ones was 0.09.

The analysis of the graphs in Figure 2, Figure 3, Figure 4 and Figure 5 shows that the effectiveness of boric acid was not only due to lowering the frictional resistance (lower coefficients of friction) but also due to increasing the durability of the lubricating film, which is especially important in the case of sheet metal forming processes, where high normal pressures often occur.

**Figure 6:**
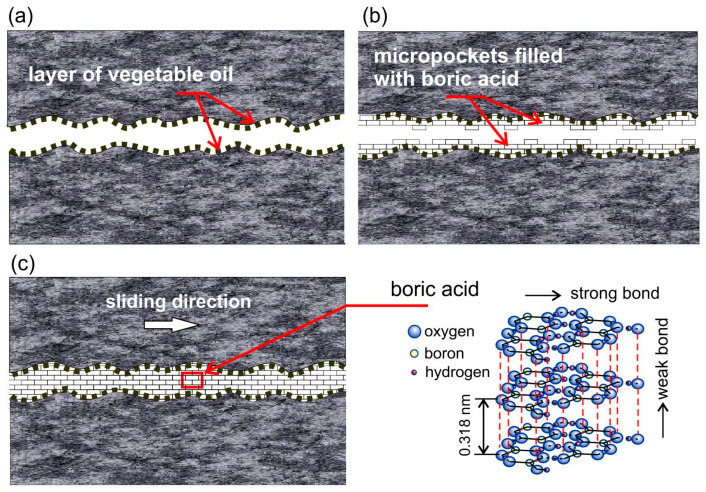
Scheme of tribological mechanism of boric acid as lubricant: (**a**) layer of vegetable oil; (**b**) deposition of boric acid particles in surface depressions (oil layer increases the adhesion of boric acid); (**c**) sliding between weakly bonded layers of boric acid.

A scheme of the tribological mechanism of boric acid as a lubricant is shown in Figure 6. The method of applying boric acid to the surface of the sheet according to the patent [41] includes spraying a thin layer of vegetable oil (Figure 6a) (in this case, rapeseed oil), whose task is to increase the adhesion of boric acid to the metal substrate, and then spraying powdered boric acid. During the plastic deformation of the sheet, due to traction pressures, all surface depressions are filled with particles of boric acid (Figure 6b). A thin layer of boric acid, separating the surfaces, causes the external friction between the surfaces of the sheet and the tool to be changed into internal friction in boric acid, i.e., weakly bonded layers of boric acid slide over each other (Figure 6c). This significantly reduces the *CoF*.

Even if the presence of the lubricant reduces the friction only by a small amount, lubrication is necessary anyway, because it prevents the phenomenon of sticking of the formed materials to the working surfaces of the tools, which in turn leads to a deterioration of the surface quality of the drawn parts.

**Figure 7:**
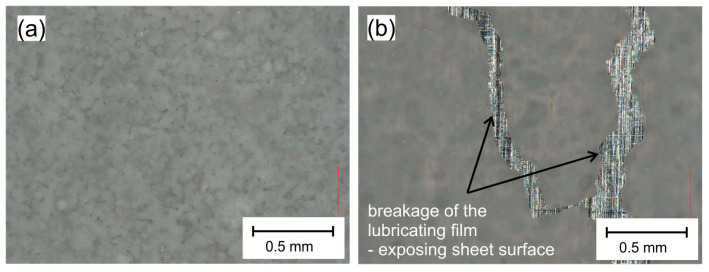
View of sheet surface with layer of lubricant No. 3 sprayed on EN AW-1050A aluminum sheet: (**a**) before strip drawing test; (**b**) after strip drawing test.

During the experimental tests, it was observed that, on some surfaces, lubricant No. 3 created a coating of low durability, easily peeling off the surface of the sheet. Usually, after spraying a 25% solution of boric acid in methyl alcohol on the sheet and evaporating the alcohol, a homogeneous coating formed on the surface of the sheets (Figure 7a). Unfortunately, in the case of EN AW-1050A aluminum and EN X5CrNi18-10 steel sheets, the coating tended to delaminate under the action of even small forces. When these sheets were drawn between the steel anvils, the coating broke (Figure 7b), and the exposed sheet materials tended to stick to the surface of the tools. Such behavior of the lubricant may not be conducive to lowering the coefficient of friction in actual forming processes where the durability of the lubricating layer is of paramount importance, particularly when forming sheets with the dominant share of tensile forces and forming deep drawn parts, especially in multistage forming. In the case of forming such sheets, a better solution is to use rapeseed oil with the addition of boric acid applied to the surface of the sheet in accordance with the patent [41] (lubricant No. 2).

Further evaluation of the lubricant’s effectiveness was carried out on the basis of the laboratory Erichsen cupping test, which was dominated by biaxial tensile stresses, and forming cylindrical drawn parts, where both stretching and drawing occurred.

**Figure 8:**
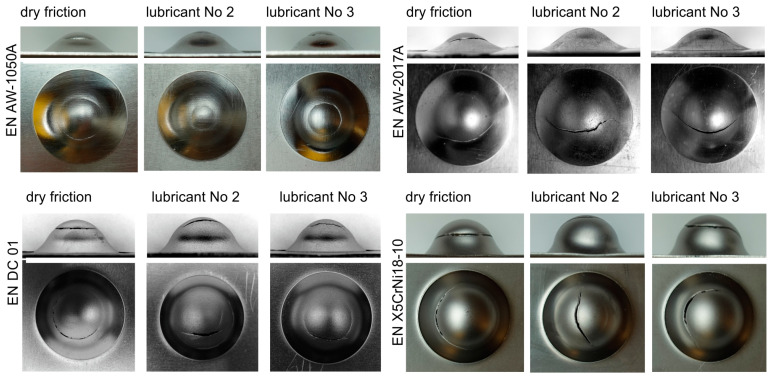
View of cups after Erichsen cupping test.

The test results obtained in the Erichsen cupping test are presented in Figure 8 and in Table 3. The tests were carried out without lubrication and with lubricants No. 2 and No. 3, i.e., lubricants that most effectively reduced the frictional resistance during the strip drawing test and eliminated sticking of the deformed material on the working surfaces of the tools.

During sheet metal forming, friction between the sheet and the working surface of the tool plays a significant role, as it affects the strain distribution in the drawn parts and, thus, determines the height that can be obtained without fracture. During the Erichsen cupping test, the sheet is thinned as a result of biaxial stretching. Getting deeper cups is possible thanks to a more even distribution of strains. The friction between the sheet and the punch counteracts the thinning of the sheet in the area of its pole (it prevents the material points of the sheet from moving away from the drawn part pole); therefore, the greatest thinning and possible cracking of the drawn part appears at a distance from its pole and runs in the circumferential direction (Figure 8—column with dry friction). The greater the frictional force between the punch and the formed sheet (poor lubrication), the farther from the axis of the drawn part the thinning of the sheet occurs, resulting in a fracture. With the reduction in frictional resistance or its absence, the greatest thinning/fracture occurs in the central part of the drawn part near its pole (Figure 8—columns with lubricant No. 2 and No. 3).

For each of the tested sheets, the use of lubricant with boric acid increased the height of the drawn part at the onset of fracture, and the change in fracture location indicated a significant reduction in friction. In addition, lubrication is necessary because each subsequent forming operation increases the risk of sticking of the formed material to the tool surface and the tendency to gall.

**Figure 9:**
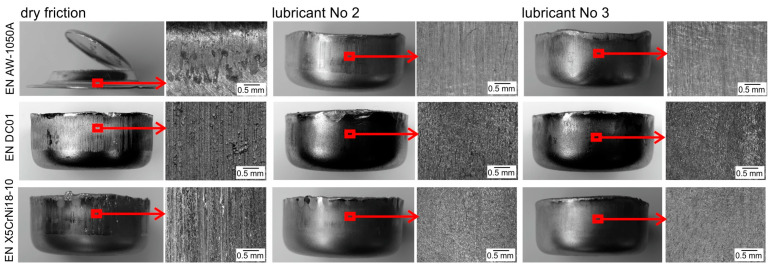
View of formed cylindrical cups.

The results of forming cylindrical drawn parts are shown in Figure 9 and Table 4. The tests were carried out using a double-action hydraulic press equipped with a tool consisting of a cylindrical punch with a diameter of 33.7 mm, a die with an inner diameter of 36 mm, and a blank holder with an inner diameter of 37 mm. As in the case of the Erichsen cupping test, the tests were carried out in dry friction conditions and in the presence of No. 2 and No. 3 lubricants. Due to the low drawability of the EN AW-2017A sheet, all the samples were damaged (circumferential crack, detachment of the drawn part bottom), regardless of whether they were formed under the conditions of technically dry friction or in the presence of lubricants.

The tests showed a positive effect of lubricants with the addition of boric acid on the reduction in the force needed to form the drawn parts compared to forming without lubricant. Slightly better results were obtained for lubricant No. 2, i.e., rapeseed oil with the addition of boric acid.

**Figure 10:**
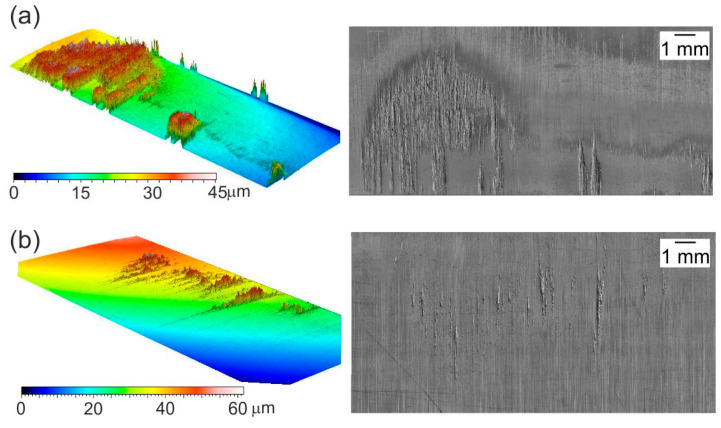
Topography of polished tool surfaces made of NC10 steel contacting with (**a**) aluminum EN AW-2017A, and (**b**) steel EN DC01.

When the tests were carried out in conditions of technically dry friction, or when the lubricating film was broken and there was a direct contact of the formed sheet with the tool, buildups of the formed material occurred at the working surfaces of the tools. These buildups made it difficult to form further drawn parts, causing scratches and dents, which were impossible to remove in subsequent operations. The surface topography of steel tools with visible traces of adhesive wear in the form of buildups of the formed material is shown in Figure 10.

**Figure 11:**
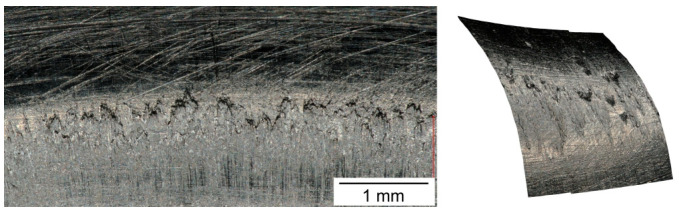
Buildups of formed material on drawing die after forming cylindrical cup made of EN DC01 steel when lubricating film was broken.

Lack of lubrication due to fracture of the lubricating film caused the deterioration of the surface quality of the drawn parts. Numerous scratches were observed on the side surface (wall) of the drawn parts, which were the result of the buildups on the fillet radius of the drawing die, as shown in Figure 11.

**Figure 12:**
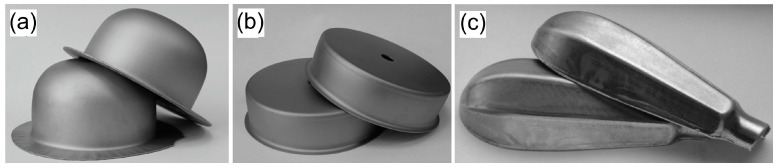
Steel drawn parts formed with lubrication: (**a**,**b**) EN DC01 steel with lubricant No. 3; (**c**) EN X5CrNi18-10 steel with lubricant No. 2.

A durable lubricant layer effectively separates contact surfaces and prevents creation of buildups, thanks to which good-quality drawn parts without scratches and dents can be obtained (Figure 12).

The use of boric acid as a lubricant enables not only the formation of drawn parts having smooth surface without scratches, but also, which is very important from the environmental protection perspective, the elimination of the previously used mineral oil-based lubricant from the technological process, which is difficult to wash and requires expensive disposal.

## 4. Conclusions

On the basis of the experiments carried out, the following conclusions can be drawn:

- The role of technological lubricants in sheet metal forming processes is not limited to reducing frictional resistance only. Technological lubricants must also separate contact surfaces such that no buildups of the formed material occur at the forming tools.
- The commonly used strip drawing test is insufficient to assess the effectiveness of lubricants in sheet metal forming processes. Tests of sheet drawability, such as the Erichsen cupping test, may be helpful.
- The tests carried out showed that the effectiveness of lubrication is not unambiguous for all frictional pairs, but depends on the type of the deformed material, the method of preparing the working surface of the tool, and the method of applying lubricant; therefore, lubrication should be selected individually for a given technological process.
- Tests showed that the coating of 25% boric acid solution in methyl alcohol (lubricant No. 3), after alcohol evaporation, is not very flexible, and that, when the sheet material becomes plasticized, i.e., when the sheet material is stretched, the coating cracks and peels off the sheet, not ensuring full protection against direct contact of surfaces. Thus, better results were achieved with the use of No. 2 lubricant, i.e., boric acid sprayed at the layer of rapeseed oil applied to the sheet. This was especially observed in sheet metal forming of EN AW-1050A.
- An effective way to apply boric acid to the sheet before forming is to spray the powdered acid onto a thin layer of oil previously applied to the sheet.
- Boric acid as a lubricant is an excellent alternative to graphite, whose main disadvantage is the occurrence of difficult-to-remove dirt on the surface of the drawn parts. Boric acid can be used in the form of colorless crystals.
- The preparation of the working surfaces of the tools has a significant impact on the amount of frictional resistance between the rubbing surfaces. Greater surface roughness of the tools after grinding is conducive to the formation of lubricating micropockets, which, when filled with boric acid, cause the mutual movement of the rubbing surfaces in the layer of boric acid along the planes of easy sliding. This lubrication mechanism effectively helps to reduce the coefficient of friction.
- The roughness of the working surface of the tools affects the formation of the so-called lubrication pockets. More favorable lubrication conditions were observed in the case of ground tools, for which the decrease in coefficient of friction was within the range of 80–90% in relation to nonlubricated surfaces.
- Lubrication always facilitates the flow of the deformed material, resulting in deeper drawn parts. In the case of the Erichsen cupping test, the effect of lubrication was the most visible for EN X5CrNi18-10 steel, for which lubricant No. 2 caused an increase in the depth of the cups by about 25%, and lubricant No. 3 caused an increase by about 15% compared to forming without lubrication.
- The use of lubrication resulted in a reduction in the forming force by about 30–40% compared to forming without lubrication. The method of applying the boric acid to the sheet had no significant effect on the value of the forming force.

## Figures and Tables

**Table 1:** Mechanical properties.

Material	Offset Yield Point R_p0.2_, MPa	Tensile Strength R_m_, MPa	Elongation A_10_, %
EN AW-2017A	282	442	16
EN AW-1050A	95	110	9
EN DC01	181	320	33
EN X5CrNi18-10	279	654	60

**Table 3:** Results of Erichsen cupping test.

Material	Erichsen Index (Cupping Depth at Moment of Fracture), mm		
	Technically Dry Friction	Lubricant No. 2	Lubricant No. 3
EN AW-1050A, t = 1 mm	7.8 ± 0.1	8.5 ± 0.1	8.4 ± 0.1
EN AW-2017A, t = 1 mm	6.9 ± 0.1	7.7 ± 0.1	7.7 ± 0.1
EN DC01, t = 0.9 mm	11.2 ± 0.1	11.8 ± 0.1	11.4 ± 0.1
EN X5CrNi18-10, t = 1 mm	13.1 ± 0.1	16.5 ± 0.1	15.0 ± 0.1

**Table 4:** Results of cylindrical cups forming.

Material	Maximum Forming Force *F_f_*, kN		
	Dry Friction	Lubricant No. 2	Lubricant No. 3
EN AW-2017A	Fracture	Fracture	Fracture
EN AW-1050A, t = 1 mm, *F_b-h_* = 1 kN *F_b-h_* = 0.5 kN	Fracture of drawn part, sheet wrinkling	6.2 ± 0.2	6.5 ± 0.2
EN DC01, t = 0.9 mm, *F_b-h_* = 2.5 kN	31.5 ± 1	18.7 ± 1	18.8 ± 1
EN X5CrNi18-10, t = 1 mm, *F_b-h_* = 2.5 kN	53.0 ± 1	39.0 ± 1	41.0 ± 1

## Data Availability

The data presented in this study are available on request from the corresponding author.

## References

[1] Cooper D.R., Rossie K.E., Gutowski T.G. (2017). The energy requirements and environmental impacts of sheet metal forming: An analysis of five forming processes. J. Mater. Process. Technol..

[2] Cooper D.R., Rossie K.E., Gutowski T.G. (2017). An environmental and cost analysis of stamping sheet metal parts. J. Manuf. Sci. Eng..

[3] Mori K. (2018). Stamping Processes for Lightweighting Automobiles. J. Jpn. Soc. Precis..

[4] Dewang Y., Sharma V. (2023). Sheet metal shrink flanging process: A critical review of current scenario and future prospects. Mater. Manuf. Process..

[5] Abe Y., Sugiura K., Mori K. (2020). Ironing limit of aluminium alloy cups with lubricants containing nanoparticles and tool steel die. Procedia Manuf..

[6] Abe Y., Daodon W., Takahashi N., Mori K. (2016). Improvement of seizure resistance by roughening surface of stainless steel drawn cup in ironing using die having lubricant pockets. Prod. Eng..

[7] Tan C.J., Aslian A., Abe Y., Mori K. (2016). Improved seizure resistance of ultra-high-strength steel ironedcups with a lubricant containing SiO_2_ nanoparticles. Int. J. Adv. Manuf. Technol..

[8] Wieckowski W., Dyja K. (2017). The effect of the use of technological lubricants based on vegetable oils on the process of titanium sheet metal forming. Arch. Met. Mater..

[9] Wieckowski W., Motyka M., Adamus J., Lacki P., Dyner M. (2022). Numerical and Experimental Analysis of Titanium Sheet Forming for Medical Instrument Parts. Materials.

[10] Jivan R.B., Eskandarzade M., Bewsher S.R., Leighton M., Mohammadpour M., Saremi-Yarahmadi S. (2022). Application of solid lubricant for enhanced frictional efficiency of deep drawing process. Proc. Inst. Mech. Eng. Part C J. Mech. Eng. Sci..

[11] Arinbjarnar U., Moghadam M., Nielsen C.V. (2021). Application of calcium carbonate as green lubricant additive in sheet metal forming. Key Eng. Mater..

[12] Trzepieciński T., Szewczyk M., Szwajka K. (2022). The use of non-edible green oils to lubricate DC04 steel sheets in sheet metal forming process. Lubricants.

[13] Trzepiecinski T. (2022). Polynomial multiple regression analysis of the lubrication effectiveness of deep drawing quality steel sheets by eco-friendly vegetable oils. Materials.

[14] Kabir M.A., Higgs C.F., Lovell M. (2009). Development of a novel green lubricant for sheet metal forming Operation. International Joint Tribology Conference (IJTC) IJTC2007-44289.

[15] Shankar S., Manikandan M., Raja G., Priyadharashini G.S. (2021). Experimental studies on viscosity, thermal and tribological properties of vegetable oil (kapok oil) with boric acid as an additive. Micro Nano Lett..

[16] Kumar R., Gautam R.K. (2022). Tribological investigation of sunflower and soybean oil with metal oxide nanoadditives. Biomass Conv. Bioref..

[17] Trzepiecinski T. (2020). Tribological Performance of Environmentally Friendly Bio-Degradable Lubricants Based on a Combination of Boric Acid and Bio-Based Oils. Materials.

[18] Sikdar S., Rahman M.H., Menezes P.L. (2022). Synergistic Study of Solid Lubricant Nano-Additives Incorporated in canola oil for Enhancing Energy Efficiency and Sustainability. Sustainability.

[19] Reeves C.J., Menezes P.L. (2016). Advancements in eco-friendly lubricants for tribological applications: Past, present, and future. Ecotribology.

[20] Bartz W.J. (2006). Ecotribology: Environmentally acceptable tribological practices. Tribol. Int..

[21] Bachchhav B.D. (2018). Challenges in Formulating Vegetable Based Metalworking Lubricants: A Review. Proceedings of the TRIBOINDIA-2018 an International Conference on Tribology.

[22] Liu Y., Binks B.P. (2021). Foams of vegetable oils containing long-chain triglycerides. J. Colloid Interf. Sci..

[23] Deshmukh P., Lovell M., Sawyer W.G., Mobley A. (2006). On the friction and wear performance of boric acid lubricant combinations in extended duration operations. Wear.

[24] Chowdary K., Kotia A., Lakshmanan V., Elsheikh A.H., Ali M.K.A. (2021). A review of the tribological and thermo-physical mechanisms of bio-lubricants based nanomaterials in automotive applications. J. Mol. Liq..

[25] Ye Q., Liu S., Xu F., Zhang J., Liu S.J., Liu W.M. (2020). Nitrogen-phosphorus codoped carbon nanospheres as lubricant additives for antiwear and friction reduction. ACS Appl. Nano Mater..

[26] Manu B.R., Gupta A., Jayatissa A.H. (2021). Tribological Properties of 2D Materials and Composites—A Review of Recent Advances. Materials.

[27] Liu L., Zhou M., Jin L., Li L., Mo Y., Su G., Li X., Zhu H., Tian Y. (2019). Recent advances in friction and lubrication of graphene and other 2D materials: Mechanisms and applications. Friction.

[28] Li H., Wang J., Gao S., Chen Q., Peng L., Liu K., Wei X. (2017). Superlubricity between MoS2 Monolayers. Adv. Mater..

[29] Vazirisereshk M.R., Martini A., Strubbe D.A., Baykara M.Z. (2019). Solid lubrication with MoS2: A review. Lubricants.

[30] Pena-Parás L., Maldonado D., Taha-Tijerina J., Martínez L., Kharissova O., Kharisov B. (2019). Eco-friendly nanoparticle additives for lubricants and their tribological characterization. Handbook of Ecomaterials.

[31] Reeves C.J., Menezes P.L. (2017). Evaluation of boron nitride particles on the tribological performance of avocado and canola oil for energy conservation and sustainability. Int. J. Adv. Manuf. Technol..

[32] Rao K.P., Xie C.L. (2006). A comparative study on the performance of boric acid with several conventional lubricants in metal forming processes. Tribol. Int..

[33] Shekhar H., Dumpala R. (2021). Overcoming friction and steps towards superlubricity: A review of underlying mechanisms. Appl. Surf. Sci. Adv..

[34] Hod O., Meyer E., Zheng Q., Urbakh M. (2018). Structural superlubricity and ultralow friction across the length scales. Nature.

[35] Luo J., Zhou X. (2020). Superlubricitive engineering-Future industry nearly getting rid of wear and frictional energy consumption. Friction.

[36] De Barros Bouchet M., Martin J., Avila J., Kano M., Yoshida K., Tsuruda T., Bai S., Higuchi Y., Ozawa N., Kubo M. (2017). Diamond-like carbon coating under oleic acid lubrication: Evidence for graphene oxide formation in superlow friction. Sci. Rep..

[37] Zhai W., Zhou K. (2019). Nanomaterials in Superlubricity. Adv. Funct. Mater..

[38] Li H., Zhang Y., Li C., Zhou Z., Nie X., Chen Y., Cao H., Liu B., Zhang N., Said Z. (2022). Extreme pressure and antiwear additives for lubricant: Academic insights and perspectives. Int. J. Adv. Manuf. Technol..

[39] Sen N., Sirin S., Kivak T., Civek T., Seçgin O. (2022). A new lubrication approach in the SPIF process: Evaluation of the applicability and tribological performance of MQL. Tribol. Int..

[40] Lovell M.R., Higgs C.F., Deshmukh P., Mobley A. (2006). Increasing formability in sheet metal stamping operations using environmentally friendly lubricants. J. Mater. Process. Technol..

[41] Adamus J., Więckowski W., Dyja K., Podlewski J. (2014). Method of Applying Lubricant on Surface of Sheet Made of Hard to Deform Material before Cold Sheet Metal Forming.

[42] Więckowski W., Adamus J., Dyner M. (2020). Sheet metal forming using environmentally benign lubricant. Arch. Civ. Mech. Eng..

[43] (2014). Metallic Materials—Sheet and Strip—Erichsen Cupping Test.

[44] (2022). Aluminium and Aluminium Alloys. Chemical Composition and Form of Wrought Products. Part 3: Chemical Composition and Form of Products.

[45] (2009). Cold Rolled Low Carbon Steel Flat Products for Cold Forming. Technical Delivery Conditions.

[46] (2015). Stainless Steels. Part 3: Technical Delivery Conditions for Semi-Finished Products, Bars, Rods, Wire, Sections and Bright Products of Corrosion Resisting Steels for General Purposes.

[47] (2002). Tool Steels.

